# Targeting Protein-Bound Uremic Toxins: A Dual Approach with Medium Cut-Off Membrane Dialysis and a Dietary Intervention—A Randomized Controlled Study

**DOI:** 10.3390/jcm15093228

**Published:** 2026-04-23

**Authors:** Tjaša Herič, Tjaša Vivoda, Špela Bogataj, Aljoša Kuzmanovski, Joško Osredkar, Joanna Giebułtowicz, Jernej Pajek

**Affiliations:** 1 Department of Nephrology, University Medical Centre Ljubljana, 1000 Ljubljana, Slovenia; tjasa.vivoda@kclj.si (T.V.); spela.bogataj@kclj.si (Š.B.); aljosa.kuzmanovski@kclj.si (A.K.); jernej.pajek@mf.uni-lj.si (J.P.); 2 Faculty of Medicine, University of Ljubljana, 1000 Ljubljana, Slovenia; 3 Faculty of Sport, University of Ljubljana, 1000 Ljubljana, Slovenia; 4 Institute of Clinical Chemistry and Biochemistry, University Medical Centre Ljubljana, 1000 Ljubljana, Slovenia; josko.osredkar@kclj.si; 5 Department of Bioanalysis and Drug Analysis, Medical University of Warsaw, 02-091 Warsaw, Poland; joanna.giebultowicz@wum.edu.pl

**Keywords:** fiber supplementation, indoxyl sulfate, medium cut-off dialyzer, p-cresyl sulfate, protein-bound uremic toxins

## Abstract

**Background/Objectives**: Protein-bound uremic toxins (PBUTs), particularly p-cresyl sulfate (PCS) and indoxyl sulfate (IS), are associated with cardiovascular toxicity and increased mortality. Conventional hemodialysis (HD) removes PBUTs poorly, and the efficacy of medium cut-off (MCO) dialyzer membranes remains uncertain. Furthermore, PBUT production is influenced by gut microbial metabolism and can be modified through diet. We hypothesized that MCO dialysis would provide superior clearance of PCS and IS compared with online hemodiafiltration (OL-HDF), and that combining MCO dialysis with increased dietary fiber and short-chain fatty acid (SCFA) intake would further reduce PBUT levels. **Methods**: In this prospective randomized trial, 62 maintenance HD patients underwent a 2-week wash-in period with high-flux HD (HF-HD) and were then randomized to MCO-HD (EXP) or OL-HDF (CON). After a 4-week intervention with the assigned dialysis modality, both groups continued with the same dialysis treatment and received an 8-week dietary intervention consisting of 19 g/day fiber and 1 g/day sodium propionate. The study concluded with a 4-week wash-out period on HF-HD. Primary outcomes were total serum PCS and IS levels measured at four timepoints. **Results**: Fifty-two patients completed the study. No significant changes in PCS or IS were observed after the dialysis-only intervention. PCS levels remained stable throughout the study. When the aligned dialysis regimen was combined with the dietary intervention, IS levels were significantly lower in the CON than in the EXP group (31.5 ± 10.3 vs. 42.0 ± 15.8 µmol/L; *p* = 0.006), with a partial rebound after wash-out in the CON group (39.6 ± 20.9 µmol/L; *p* = 0.003). **Conclusions**: While MCO-HD and OL-HDF had a similar effect on serum PCS and IS concentrations, only OL-HDF combined with the dietary intervention significantly reduced IS levels.

## 1. Introduction

Chronic kidney disease (CKD) represents a growing global health burden, with a prevalence of approximately 14%. It ranks as the ninth leading cause of death worldwide and is a major contributor to cardiovascular mortality [1]. Advanced stages of CKD requiring kidney replacement therapy further increase this burden, affecting more than 3.5 million individuals worldwide [2].

Inadequate clearance of certain uremic toxins plays a key role in adverse clinical outcomes in patients undergoing chronic hemodialysis (HD) [3]. The removal of medium-sized and protein-bound uremic toxins (PBUTs) remains limited with high-flux HD (HF-HD) [3,4]. Online hemodiafiltration (OL-HDF) with a high-flux membrane is the current gold standard for chronic HD, offering superior clearance of medium-sized molecules (molecular weight [MW] > 500 Da) compared to HF-HD [5]. However, the clearance of PBUTs—uremic toxins primarily bound to serum albumin (MW~66,500 Da)—remains inadequate even with OL-HDF [6,7].

Protein-bound uremic toxins (PBUTs), particularly p-cresyl sulfate (PCS) and indoxyl sulfate (IS), have been implicated in increased cardiovascular morbidity, mortality and all-cause mortality in patients with CKD and those receiving chronic HD [8,9,10,11,12,13,14]. Given this strong link between uremic toxin accumulation and cardiovascular risk, strategies aimed at reducing toxin burden may contribute to improving outcomes in this population.

Medium cut-off (MCO) membranes have been developed to enhance the clearance of larger molecules while preserving serum albumin concentrations [15]. Their main advantages are lower water requirements and simpler implementation compared to OL-HDF, which requires over 23 L of substitution volume to improve survival [16]. Compared to HF-HD, MCO dialysis more effectively removes medium-sized molecules [15,17]. However, evidence on its efficacy in clearing PBUTs, namely PCS and IS, remains scarce, indicating no improvement in their clearance compared with OL-HDF [18].

In addition to dialysis, dietary factors influence serum PBUT levels. PCS and IS, two major PBUTs, are derived from gut microbial metabolism of dietary proteins, whereby intestinal bacteria convert the aromatic amino acids tyrosine and phenylalanine into p-cresol and tryptophan into indole, which are subsequently sulfated to form PCS and IS [19]. In CKD, uremia contributes to gut dysbiosis through multiple mechanisms, including increased intestinal urea levels with subsequent ammonia production and pH alterations, dietary patterns in CKD patients characterized by reduced fiber intake, and disruption of the intestinal barrier, all of which promote a shift toward proteolytic bacterial metabolism and increased generation of toxin precursors [20,21]. Their accumulation is further exacerbated by markedly reduced renal clearance and limited removal by dialysis due to their high protein binding, resulting in serum PCS and IS concentrations in HD patients that can be up to 100 times higher than in healthy individuals [22]. Dietary interventions, particularly increased fiber intake, have been shown to reduce serum PCS and IS levels in some randomized controlled trials [23,24,25,26]. Additionally, dietary supplementation with sodium propionate, a short-chain fatty acid (SCFA), has been shown to effectively reduce circulating levels of PCS and IS in patients undergoing chronic HD [27].

The present study compares the serum concentrations of PCS and IS achieved after 4 weeks of treatment with MCO dialysis versus OL-HDF. In the study extension phase, it also investigates how the combination of increased dietary fiber intake and sodium propionate supplementation with each dialysis modality affects the levels of these two PBUTs. We hypothesized that MCO dialysis provides superior clearance and lower serum values of PCS and IS compared to OL-HDF. Furthermore, we proposed that the combination of MCO dialysis with a dietary intervention would result in a greater reduction in PCS and IS than OL-HDF.

## 2. Materials and Methods

Between February 2021 and June 2022, a prospective, randomized controlled study was conducted on 62 chronic HD patients at the University Medical Centre Ljubljana, Slovenia. The study adhered to the ethical principles of the Declaration of Helsinki and was approved by the National Medical Ethics Committee of the Republic of Slovenia (Approval No. 0120-430/2019/12, dated 22 October 2019). The study was registered at ClinicalTrials.gov (Identifier: NCT04247867). The study protocol has been previously published [18].

### 2.1. Subjects

Eligible participants were prevalent adult patients receiving chronic HD for at least 12 weeks, with a functioning arteriovenous (AV) fistula or graft as permanent vascular access, who provided informed written consent and were able to adhere to the study diet. Patients were excluded if their baseline serum albumin was below 32 g/L, if they had undergone transplantation, or if, within 4 weeks prior to study initiation or during the study, they had an active infection, a newly confirmed malignancy, or a cardiovascular or cerebrovascular event or required hospitalization, as this was considered indicative of clinical instability.

### 2.2. Study Design

**Figure 1:**
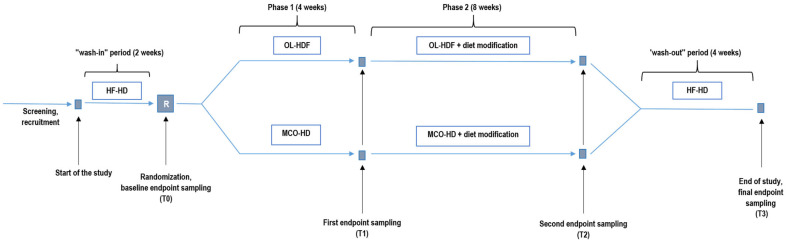
Study design and timeline.

All patients underwent a two-week wash-in period with HF-HD. Patients were then randomized in a 1:1 ratio into experimental (EXP) and control (CON) groups using a computer-generated random sequence. In Phase 1 (4 weeks), the EXP group received MCO dialysis, while the CON group underwent OL-HDF. In Phase 2 (8 weeks), all patients continued with the allocated dialysis modality and in addition received a dietary intervention designed to increase daily fiber intake by 19 g—achieved by consuming whole-grain flaxseed biscuits (9 g/day) and a powdered supplement containing psyllium and inulin (10 g/day)—along with 1 g/day of sodium propionate, under the guidance of a clinical dietitian. The intervention was designed to assess the combined effect of increased dietary fiber intake and SCFA supplementation. The study concluded with a four-week wash-out period using HF-HD to assess the persistence of intervention effects after discontinuation. Blood samples were collected after the wash-in period (T0), after Phase 1 (T1), after Phase 2 (T2), and finally after the wash-out period (T3) (see Figure 1).

Baseline clinical and demographic characteristics were recorded, including overhydration assessed by bioimpedance spectroscopy using a Body Composition Monitor (BCM; Fresenius Medical Care, Bad Homburg, Germany) at T0, prior to the midweek HD session.

Blood samples were obtained from the arterial line of a functioning AV fistula or graft immediately (within a time frame of 2–3 min) before the start of the midweek HD session. The midweek session was defined as the second dialysis session of the week (Wednesday for patients on a Monday–Wednesday–Friday schedule and Thursday for those on a Tuesday–Thursday–Saturday schedule). Approximately 5 mL of blood was collected into serum separator tubes, allowed to clot for 30 min at room temperature, and centrifuged at 1500× *g* for 30 min. Serum aliquots were stored at −80 °C until batch analysis.

### 2.3. Outcomes

#### 2.3.1. Primary Outcomes

Total serum PCS and IS concentrations were determined using validated high-performance liquid chromatography coupled with tandem mass spectrometry (LC-MS/MS). Serum samples were spiked with an internal standard (600 ng mL^−1^), mixed with methanol (1:1:8, *v*/*v*), incubated at −20 °C for 20 min, and centrifuged (9300× *g*, 10 min, 4 °C). Supernatants were diluted sixfold with mobile phase and analyzed by LC–MS/MS. An Agilent 1260 Infinity system coupled to a QTRAP 4000 mass spectrometer was used. Nitrogen served as curtain, nebulizer, and auxiliary gas; ion spray voltage was 4500 V and source temperature 600 °C. Separation was performed on a Kinetex C18 column (40 °C) using water and methanol with 0.1% formic acid at 0.5 mL min^−1^ under gradient conditions. Injection volume was 10 µL. Detection was conducted in multiple reaction monitoring mode with two transitions per analyte, and optimized declustering potential, collision energy, entrance potential, and collision cell exit potential voltages were applied for IS, IS-d_4_, PCS, and PCS-d_7_.

#### 2.3.2. Secondary Outcomes

Secondary outcomes included serum concentrations of albumin and selected small- (potassium, phosphate, urea, creatinine, trimethylamine-N-oxide [TMAO]) and middle-sized (β2-microglobulin [B2M]) uremic toxins. All analytes were quantified using automated laboratory methods on Abbott analyzers, except for TMAO, which was analyzed using LC-MS/MS. Additionally, the Dialysis Symptom Index (DSI), another secondary endpoint, was measured at all four timepoints. The DSI is a validated patient-reported questionnaire consisting of 30 questions used to assess the frequency and severity of dialysis-related physical and emotional symptoms over the previous week [28]. Each symptom is first recorded as present or absent, and, if present, its severity is rated on a 5-point Likert scale. The overall DSI score reflects cumulative symptom burden, with higher scores indicating a greater number and/or severity of symptoms. Patients received the questionnaire at each study timepoint and returned the completed questionnaire at their subsequent HD session.

### 2.4. Dialysis Procedure

Throughout the study, each patient had constant blood flow rate and HD duration. The dialysate flow rate was maintained at 500 mL/min for all patients throughout the study. Convection volume was targeted at ≥23 L per session for OL-HDF and varied depending on the net ultrafiltration. The technical characteristics of the dialysis procedure are presented in Table 1.

### 2.5. Dietary Intervention and Adherence

Before, during, and after the dietary intervention, participants completed a 3-day food diary, which was used to calculate fiber intake. During Phase 2 (dietary intervention), supplement fiber intake was estimated using a questionnaire assessing adherence to the prescribed intake (ranging from 0 to 19 g per day). To improve compliance, participants were instructed to consume one biscuit and one portion of the powdered supplement during dialysis sessions, under the supervision of a dialysis nurse. On dialysis days, they were also provided with the same products to take home for consumption on non-dialysis days.

### 2.6. Sample Size Calculation

Sample size calculation was informed by previously published data on serum IS concentrations reported by Esgalhado et al. [26]. Based on these data, a difference of approximately 3.8 mg/L between groups was considered clinically relevant. In the absence of directly applicable estimates of variability, a standard deviation (SD) of 5 mg/L was assumed, resulting in an estimated effect size of 0.76. With a significance level (α) of 0.05 and statistical power (1 − β) of 0.80, the estimated sample size was 46 participants. Accounting for an anticipated dropout rate of 10%, a total of 52 participants were required.

### 2.7. Statistical Analysis

All statistical analyses were performed using SPSS software, version 27.0 (SPSS Inc., Chicago, IL, USA). Normality of distribution was assessed using the Shapiro–Wilk test. Data are presented as mean ± SD unless otherwise specified. The two study groups (EXP and CON) were treated as independent between-subject factors, while timepoints (T0, T1, T2, T3) were considered within-subject repeated measures. All outcome variables were categorized as dependent variables. Repeated measures analysis of variance (RM ANOVA) was used to assess changes over time and between groups for variables with a normal distribution. Where statistically significant main effects or interactions were identified, post hoc comparisons were performed using paired-sample *t*-tests. For non-normally distributed data, comparisons over time and between groups were conducted using the Generalized Estimating Equation (GEE) method, with Friedman’s post hoc test applied for within-group comparisons, and the Mann–Whitney U test used for between-group comparisons at individual timepoints. All statistical tests were two-tailed, and a *p*-value of <0.05 was considered statistically significant. The final analysis was performed on a per-protocol basis, including only patients who completed the study.

## 3. Results

**Figure 2:**
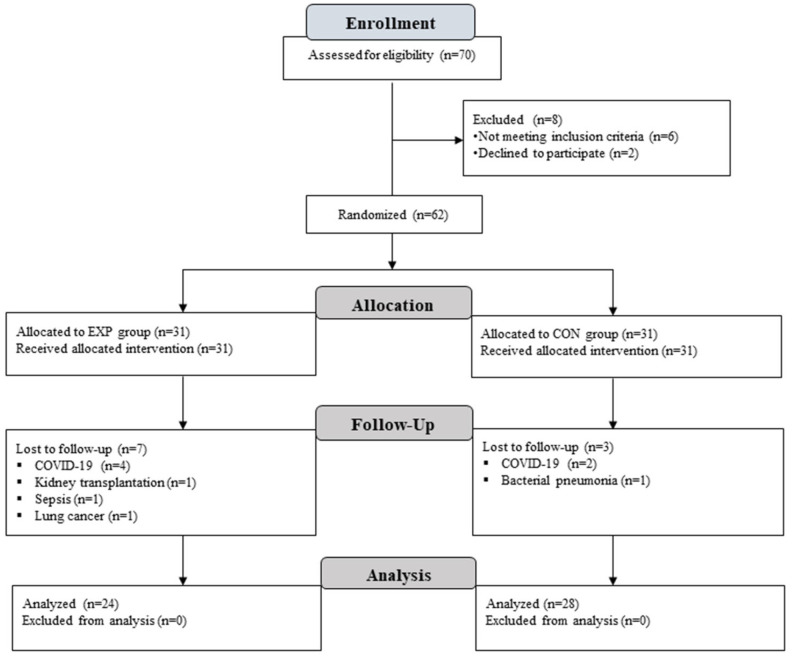
Flowchart.

Out of 62 randomized patients, 52 completed the study and were included in the final analysis (see Figure 2). The randomized patient groups did not differ significantly at baseline (see Table 2).

### 3.1. Primary Outcomes—P-Cresyl Sulfate and Indoxyl Sulfate

**Figure 3:**
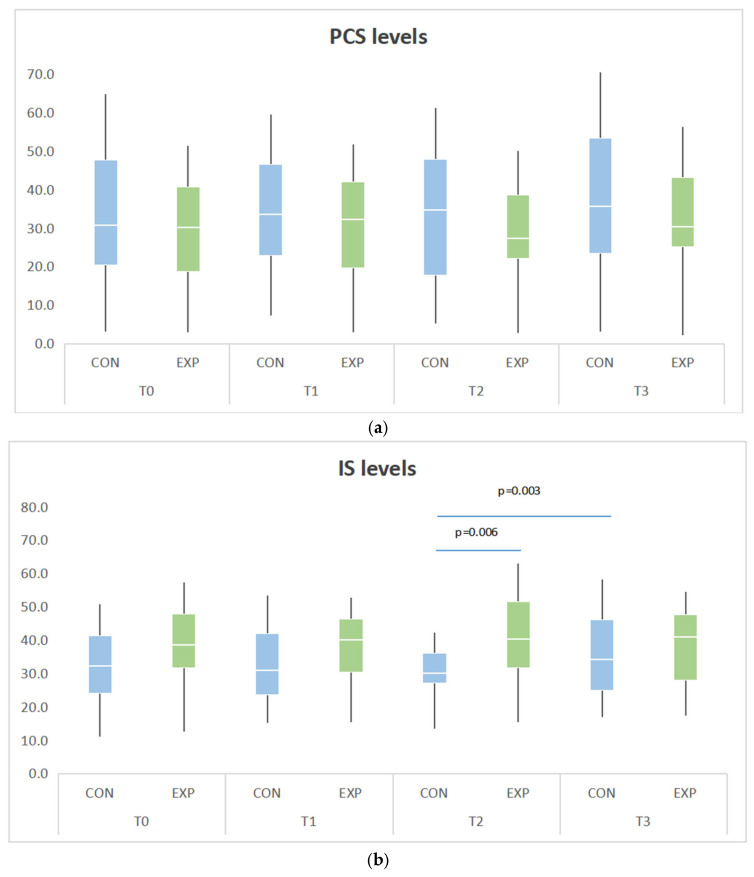
Primary outcomes: (**a**) serum p-cresyl sulfate concentrations at four timepoints (T0–T3) in the CON and EXP group; (**b**) serum indoxyl sulfate concentrations at four timepoints (T0–T3) in the CON and EXP group. Data are presented as boxplots showing the median (line), interquartile range (box), and range (whiskers).

Serum levels of PCS and IS were measured at four timepoints (T0–T3). No significant baseline differences were observed between groups for either substance (Table 3). PCS levels remained relatively stable throughout the study (see Figure 3). No significant within-group changes were observed across all timepoints (*p* = 0.079). 

IS exhibited a distinct pattern, with a significant group-by-time interaction (*p* = 0.005). While IS levels remained stable from T0 to T1 (*p* = 0.262), a significant between-group difference emerged at T2, with lower IS in the CON group compared to the EXP group (31.5 ± 10.3 vs. 42.0 ± 15.8 µmol/L; *p* = 0.006; see Table 3). Although IS decreased numerically in the CON group from T1 to T2, the change was not statistically significant. However, a significant increase occurred between T2 and T3 in the CON group (31.5 ± 10.3 vs. 39.6 ± 20.9 µmol/L, *p* = 0.003; see Figure 3), while levels in the EXP group remained stable.

### 3.2. Secondary Outcomes

A significant decrease in serum albumin was detected in the overall sample between T0 and T2 (*p* = 0.027); however, no difference was found between the groups at T2. Serum concentrations of urea, creatinine, potassium, and phosphate remained stable throughout the study, with no significant effects over time or between groups (all *p* > 0.05). Time*group interaction for B2M level was significant (*p* = 0.047), with the EXP group demonstrating lower levels (Table 3). TMAO concentrations increased modestly during the study period (*p* = 0.036). However, post hoc comparisons showed no significant changes within or between groups at any timepoint. DSI scores improved numerically during the intervention in the CON group, although this change did not reach significance within the group nor between groups. An overview of biochemical and clinical outcomes by timepoint can be found in Table 3.

**Table 3:** Laboratory values of study outcomes at different timepoints.

	CON (n = 28)				EXP (n = 24)							***p*** **-Value Between Groups Within Time Measurement**			
Variable	T0	T1	T2	T3	T0	T1	T2	T3	*p*-Value Between Measurements	*p*-Value Groups and Measurements	*p*-Value Between Groups	**T0**	**T1**	**T2**	**T3**
PCS [μmol/L]	34.2 ± 18.0	35.8 ± 18.5	33.2 ± 17.2	38.6 ± 19.9	30.6 ± 16.3	33.1 ± 17.3	32.3 ± 17.4	34.8 ± 20.8	0.079	0.798	0.556	0.455	0.583	0.849	0.510
IS [μmol/L]	34.0 ± 13.4	34.4 ± 14.0	31.5 ± 10.3	39.6 ± 20.9	38.7 ± 14.2	38.9 ± 14.6	42.0 ± 15.8	40.6 ± 16.1	0.242	**0.005**	0.174	0.230	0.262	**0.006**	0.836
Alb [g/L]	42.4 ± 2.6	41.7 ± 2.9	41.7 ± 2.8	42.0 ± 3.7	41.7 ± 2.9	42.2 ± 2.7	42.0 ± 2.8	42.9 ± 2.5	**0.013**	0.584	0.531	0.801	0.532	0.721	0.309
Urea [mmol/L]	21.1 ± 4.0	21.5 ± 5.3	22.2 ± 4.7	22.5 ± 4.8	20.2 ± 4.5	21.6 ± 5.1	20.7 ± 4.8	20.9 ± 4.4	0.285	0.425	0.363	0.432	0.961	0.249	0.216
Cr [μmol/L]	713.8 ± 125.9	711.4 ± 130.6	722.6 ± 127.3	720.9 ± 142.8	651.7 ± 128.3	658.6 ± 112.2	661.5 ± 133.9	661.8 ± 125.6	0.774	0.955	0.089	0.085	0.128	0.098	0.122
K [mmol/L]	5.4 ± 0.6	5.4 ± 0.5	5.3 ± 0.5	5.5 ± 0.7	5.3 ± 0.5	5.3 ± 0.6	5.2 ± 0.6	5.3 ± 0.5	0.195	0.804	0.318	0.560	0.544	0.471	0.205
P [mmol/L]	1.8 ± 0.6	1.8 ± 0.6	1.7 ± 0.4	1.6 ± 0.5	1.4 ± 0.3	1.6 ± 0.4	1.7 ± 0.4	1.6 ± 0.3	0.188	0.051	0.221	**0.014**	0.443	0.643	0.845
B2M [mg/L]	28.0 ± 9.1	28.3 ± 11.2	27.1 ± 9.8	28.1 ± 8.4	23.4 ± 5.5	23.9 ± 5.2	23.5 ± 4.8	24.0 ± 5.2	0.535	0.766	**0.047**	**0.037**	0.083	0.102	**0.040**
TMAO [μmol/L]	3.7 ± 1.7	4.3 ± 1.9	4.2 ± 1.7	4.3 ± 2.3	4.3 ± 2.5	4.9 ± 2.8	5.1 ± 3.5	4.6 ± 3.1	**0.036**	0.834	0.286	0.340	0.332	0.200	0.678
DSI [points]	20.0 ± 18.8	14.5 ± 13.8	13.8 ± 16.1	14.9 ± 14.2	12.7 ± 11.5	15.3 ± 13.2	14.5 ± 12.6	12.1 ± 9.8	0.362	0.120	0.598	0.151	0.845	0.886	0.484

Legend: PCS = p-cresyl sulfate; IS = indoxyl sulfate; Alb = serum albumin; Cr = serum creatinine; K = serum potassium; P = serum phosphate; B2M = β2-microglobulin; TMAO = trimethylamine N-oxide; DSI = Dialysis Symptom Index. Data are presented as mean ± SD. Bold values indicate statistically significant differences (*p* < 0.05). Changes over time and between groups were analyzed using repeated measures ANOVA or GEE, as appropriate. Reference values for healthy populations are provided for context: Alb 32–55 g/L; Cr 49–90 µmol/L; P 0.74–1.52 mmol/L; K 3.8–5.5 mmol/L; urea 2.5–6,7 mmol/L; B2M ~1.3 mg/L; IS ~5–7 µmol/L; PCS ~20–26 µmol/L; and TMAO ~3.2 µmol/L. Values for PCS, IS, TMAO, and B2M are derived from published studies and are presented as mean (±SD where available) [29,30,31]; these should be interpreted with caution, as they are not standardized and may vary across laboratories.

### 3.3. Fiber Intake

Adherence to the prescribed dietary fiber regimen was generally good in both groups. The EXP group reached 66% of total prescribed fiber supplements, while the CON group achieved 77%. Although a higher mean adherence was observed in the CON group, this difference did not reach statistical significance (*p* = 0.162). The average fiber supplement intake during the intervention phase was 14.7 ± 4.6 g in the CON and 12.3 ± 6.2 g in the EXP group. Total dietary fiber intake reached 30.3 ± 7.5 g in the CON group and 26.5 ± 6.8 g in the EXP group (*p* = 0.077; see Table 4).

## 4. Discussion

Findings from this randomized controlled trial demonstrate that MCO dialysis and OL-HDF have a comparable, yet limited, effect on serum PCS and IS concentrations. When combined with dietary intervention, including increased fiber and SCFA intake, MCO dialysis did not achieve further reduction in serum PCS and IS, whereas OL-HDF with the same dietary intervention resulted in significantly lower serum IS levels.

Our findings regarding the comparison of dialysis modalities are consistent with previous reports. The largest study conducted to date in this field, a randomized crossover trial by Kim et al. (2022) [32] involving 22 patients, found no significant differences in PCS or IS clearance between MCO-HD, post-dilution OL-HDF, and HF-HD after three weeks. A smaller crossover study of 12 patients by Biedunkiewicz et al. (2024) [33] similarly reported no differences in serum IS levels after one week when comparing various OL-HDF modes with MCO dialysis but did not evaluate PCS levels. In addition, Tiong et al. (2021) [34] also found no significant reduction in PCS or IS when comparing 12 or 24 weeks of MCO dialysis with HF-HD in a study of 89 patients; however, the study was non-randomized and did not include comparison with OL-HDF. Taken together, current evidence—to which our study adds important new data—does not support improved clearance of PCS or IS with MCO dialysis compared with OL-HDF. These findings further underscore that currently available HD modalities remain insufficient to achieve clinically meaningful reductions in PCS and IS.

The underlying reason lies in the biochemical properties of these toxins. Both PCS and IS are strongly bound to serum albumin, and their clearance in healthy kidneys relies mainly on tubular secretion [32,35], a function that dialysis cannot replicate. Evidence suggests that HF-HD and OL-HDF remove these solutes predominantly through diffusion of the free, unbound fraction [6,36,37]. Clearance is limited by the slow dissociation of PCS and IS from albumin, restricting their removal during the relatively short duration of a dialysis session and consequently leaving the protein-bound fraction largely unaffected [38]. It is important to note that our analysis was based on pre-dialysis serum concentrations, which represent a steady-state balance between toxin generation, distribution, and elimination, rather than on direct measures of dialytic clearance, such as the reduction ratio, dialysate mass removal, or clearance rates. Therefore, changes in pre-dialysis concentrations reflect overall systemic burden rather than the immediate efficiency of toxin removal during a single dialysis session.

We hypothesized that MCO dialysis, owing to its greater albumin permeability, would enhance the removal of the albumin-bound fraction and consequently lower pre-dialysis serum levels of PCS and IS. Most studies have indeed reported greater albumin losses into the dialysate with MCO membranes compared to post-dilution OL-HDF [17,32,39]. Interestingly, Kim et al. [32] reported no correlation between the dialysate albumin mass and the dialysate total IS or PCS mass. Despite the increased albumin leakage observed with MCO dialysis, the dialysate mass of total PCS and IS was not significantly different from that achieved with OL-HDF or HF-HD, and pre-dialysis plasma concentrations of PCS and IS also remained comparable across modalities. Based on these data, we can reasonably conclude that the primary mechanism of eliminating these PBUTs with MCO dialysis is through the unbound fraction, as it is with OL-HDF, which likely explains the absence of any significant differences in serum PCS and IS concentrations between the two modalities in our study.

Since the clearance of these PBUTs could not be enhanced with MCO dialysis—and further increasing membrane permeability would likely result in excessive albumin loss—an alternative and physiologically meaningful approach is to target the production of these toxins at their origin. Both PCS and IS are products of gut microbial fermentation of dietary amino acids. Increasing dietary fiber intake shifts gut microbial metabolism from proteolytic to saccharolytic pathways, thereby reducing the bacterial degradation of aromatic amino acids into uremic toxin precursors [40]. This shift is associated with increased production of SCFA, particularly butyrate, which supports colonocyte metabolism and helps maintain intestinal barrier integrity. Improved barrier function may reduce the translocation of microbial-derived toxins and their precursors into the circulation [41]. In addition, higher fiber intake is associated with shorter intestinal transit time, further limiting substrate exposure to proteolytic bacteria and reducing the generation of p-cresol and indole [40]. Several clinical studies suggest that fiber supplementation may lower circulating levels of PCS and IS, although results remain heterogeneous and may depend on the protein-to-fiber ratio [23,24,25,26,42]. In our study, we extended this concept by combining increased dietary fiber intake with sodium propionate supplementation, targeting gut microbial metabolism through complementary mechanisms—fermentable fiber to shift the microbiome away from proteolytic metabolism, and exogenous SCFA to directly support colonocyte function and barrier integrity. This pragmatic, combined approach was also chosen with patient adherence in mind, as HD patients typically face significant dietary restrictions and are often unable to implement major changes in eating habits. As both dietary components were introduced simultaneously, the study design does not allow differentiation of their individual contributions to the observed study outcomes, particularly PBUTs.

A key finding of our trial was that dietary fiber and SCFA supplementation reduced IS levels significantly, but only when combined with OL-HDF. Differences in adherence to the dietary regimen between groups represent a potential confounding factor in the interpretation of Phase 2 results. The numerically better adherence observed in the OL-HDF group may have amplified the dietary effect in that group, and could in part explain the more pronounced reduction in IS levels at T2. This variability in compliance likely reflects real-world differences in patients’ capacity to adhere to dietary modifications, rather than a systematic difference between dialysis modalities. The OL-HDF group also experienced a rebound increase in IS after discontinuation of the dietary intervention (T3), suggesting that the microbiota-related effects were not sustained. No significant effect was observed for PCS, which may reflect differences in metabolic pathways [24], interindividual variability of the microbiota and differences in habitual diet. Given that adherence is often the main barrier to the success of nutritional strategies in dialysis patients, this finding underscores the importance of structured support and follow-up. Nevertheless, patients in our study achieved generally good compliance, serum potassium remained stable, and no serious adverse events were observed, supporting the safety and feasibility of the dietary intervention in this setting. Notably, patients did not report changes in physical or emotional symptoms as assessed by the DSI, further supporting the tolerability of both interventions. The absence of symptom improvement highlights the challenge of achieving clinically perceptible benefits from biochemical changes alone in short-term studies.

The conceptual advantage of combining MCO dialysis with a dietary intervention lies in its accessibility. MCO dialysis is technically simpler and less resource-demanding than OL-HDF, while dietary supplementation with fiber and SCFA is safe, inexpensive, and widely applicable. Our study adds new evidence by testing this combined approach. Although the hypothesized greater effect was not confirmed, the feasibility and affordability of this strategy make it an attractive candidate for further research. Importantly, our findings also support the notion that current HD methods have reached a plateau in their ability to clear PBUTs. Increasing membrane permeability has not improved PCS or IS removal, whereas nutritional interventions may represent a more promising strategy to reduce toxin generation at the gut level.

### Strengths and Limitations

Our study has several notable strengths. The sample size was reasonable for a dietary intervention in HD patients and supports the reliability of our findings. The intervention was supported by a clinical dietitian, who assisted in preparing and distributing the fiber-enriched biscuits and guiding patients with dietary diaries, which likely contributed to the good level of adherence achieved. An additional strength is that, alongside biochemical measurements, the study also included a validated clinical outcome—the Dialysis Symptom Index (DSI)—providing complementary patient-reported insight into the tolerability and clinical relevance of the interventions.

Several limitations must also be acknowledged. The analysis was conducted on a per-protocol basis, which may introduce some degree of selection and attrition bias and should be considered when interpreting the generalizability of the findings. Moreover, the duration of the dietary intervention may have been insufficient to capture longer-term changes in PBUT production. The difference in weekly dialysis duration between groups represents a potential limitation. The control group received on average approximately one hour (8%) longer weekly dialysis time, which arose from randomization and reflected individual clinical needs, particularly higher ultrafiltration requirements, rather than a systematic allocation bias. Importantly, baseline serum concentrations of IS and PCS were comparable between groups at study entry (T0), indicating that this imbalance did not translate into a pre-existing difference in the primary outcomes. Moreover, dialysis duration remained constant for each patient throughout the study, ensuring that within-patient changes across timepoints reflect the effect of the study interventions rather than alterations in dialysis prescription. Finally, we did not assess PBUT levels post-dialysis or systematically measure dialysate concentrations in all patients. Further studies are needed to clarify the effect of longer dietary interventions on PCS and IS levels and to investigate the relationship of these solutes with survival and cardiovascular outcomes.

## 5. Conclusions

MCO dialysis, despite allowing greater albumin permeability, did not demonstrate an advantage over OL-HDF in reducing serum PCS or IS concentrations. The combination of MCO dialysis with increased fiber and SCFA intake was not associated with a reduction in PBUT levels, whereas OL-HDF paired with the same dietary intervention resulted in significantly lower IS concentrations. These findings highlight the limited capacity of current HD modalities to remove albumin-bound toxins and support dietary modulation—particularly increased fiber and SCFA intake—as a promising strategy for future studies aimed at reducing the PBUT burden in HD patients.

## Figures and Tables

**Table 1:** Hemodialysis treatment characteristics before and during the intervention.

Variable	All Patients (n = 52)	CON (n = 28)	EXP (n = 24)
HD duration (pre) [h/week]	12.5 ± 1.8	13.0 ± 1.4	12.0 ± 2.1
HD duration (int) [h/week]	12.5 ± 1.8	13.0 ± 1.4	12.0 ± 2.1
Dialysis modality (pre)			
Post OL-HDF	10 (19.2%)	26 (92.9%)	16 (66.7%)
HF-HD	42 (80.2%)	2 (7.1%)	8 (33.3%)
Dialyzer type (pre)			
Evodial 2.2 *	1 (1.9%)	1 (3.6%)	0
FX100 Cordiax	24 (46.1%)	14 (50.5%)	10 (41.7%)
FX60 Cordiax	3 (5.8%)	3 (10.7%)	0
FX80 Cordiax	23 (44.2%)	10 (35.7%)	13 (54.2%)
TN500	1 (1.9%)	0	1 (4.1%)
Dialyzer type (int)			
Evodial 2.2 *	1 (1.9%)	1 (3.6%)	0
FX100 Cordiax	16 (30.8%)	16 (57.1%)	0
FX60 Cordiax	0	0	0
FX80 Cordiax	11 (21.1%)	11 (39.3%)	0
TN500	13 (25.0%)	0	13 (54.2%)
TN400	11 (21.1%)	0	11 (45.8%)
Qb [mL/min] (pre)			
300	40 (76.9%)	21 (75.0%)	19 (79.2%)
250	12 (23.1%)	7 (25.0%)	5 (20.8%)
Qb [mL/min] (int)			
300	42 (80.2%)	22 (78.6%)	20 (83.3%)
250	10 (19.2%)	6 (21.4%)	4 (16.7%)
SV [L] **	N/A	27.2 ± 3.0	N/A

Legend: HD = hemodialysis; pre = before intervention; int = during intervention; post = post-dilution; Qb = blood flow; Qd = dialysate flow; SV = substitution volume per session; * one patient in the HDF group received an Evodial dialyzer due to an allergy to the FX Cordiax membrane. ** Some data were missing (n = 6); values are based on the available cases.

**Table 2:** Baseline clinical and demographic characteristics of randomized patients.

Variable	All Patients (n = 52)	CON (n = 28)	EXP (n = 24)	*p*-Value
Age [years]	65.6 ± 11.2	63.7 ± 11.8	67.8 ± 10.3	0.185
Gender—male [n (%)]	33 (63.5)	17 (60.7)	16 (66.7)	0.657
BMI [kg/m^2^]	26.9 ± 5.1	26.9 ± 5.9	27.0 ± 4.1	0.941
DW [kg]	74.7 ± 19.3	74.1 ± 23.6	75.3 ± 13.1	0.831
OH [L]	2.4 ± 1.5	2.3 ± 1.3	2.5 ± 1.7	0.649
HD vintage [years]	5.6 ± 6.2	5.7 ± 6.6	5.4 ± 5.7	0.863
CCI [points]	5.8 ± 2.2	5.5 ± 2.2	6.1 ± 2.2	0.283
Alb [g/L]	42.4 ± 2.6	42.4 ± 2.6	42.5 ± 2.6	0.801
Hb [g/L]	119.0 ± 11.2	119.6 ± 11.3	118.4 ± 11.3	0.704

Legend: HD = hemodialysis; BMI = body mass index (kg/m^2^); DW = dry weight (kg); OH = bioimpedance-measured extracellular fluid overhydration (L); HD vintage = time on maintenance hemodialysis prior to the intervention (years); CCI = Charlson Comorbidity Index (points); Alb = serum albumin (g/L); Hb = serum hemoglobin (g/L). Data are presented as mean ± SD or n (%). Continuous variables were compared using the independent samples *t*-test or Mann–Whitney U test, as appropriate, and categorical variables using the chi-square test or Fisher’s exact test, as appropriate.

**Table 4:** Daily intake of energy, protein and fiber during the phases of the dietary intervention.

	CON (n = 28)			EXP (n = 24)			*p*-Value Between Measurements	*p*-Value Groups and Measurements	*p*-Value Between Groups	*p*-Value Between Groups Within Time Measurement		
Variable	T0	T1	T2	T0	T1	T2				T0	T1	T2
Baseline fiber intake [g]	14.8 ± 5.6	15.1 ± 5.8	13.5 ± 4.5	14.9 ± 4.3	14.1 ± 4.2	13.8 ± 4.0	0.064	0.608	0.889	0.919	0.476	0.729
Fiber supplement intake [g]	/	14.7 ± 4.6	/	/	12.3 ± 6.2	/	/	/	/	/	0.135	/
Total fiber intake [g]	14.8 ± 5.6	30.3 ± 7.5	13.5 ± 4.5	14.9 ± 4.3	26.5 ± 6.8	13.8 ± 4.0	<0.001	0.084	0.314	0.919	0.077	0.729

Data are presented as mean ± SD. Changes over time and between groups were analyzed using repeated measures ANOVA or GEE, as appropriate.

## Data Availability

The data presented in this study are available on reasonable request from the corresponding author. The data are not publicly available due to privacy and ethical restrictions.

## Abbreviations

The following abbreviations are used in this manuscript:

AV	Arteriovenous
Alb	Serum albumin
B2M	β2-microglobulin
BCM	Body composition monitor
BMI	Body mass index
CCI	Charlson comorbidity index
CON	Control group
Cr	Serum creatinine
DSI	Dialysis symptom index
DW	Dry weight
EXP	Experimental group
GEE	Generalized estimating equation
Hb	Serum hemoglobin
HD	Hemodialysis
HF-HD	High-flux hemodialysis
IS	Indoxyl sulfate
K	Serum potassium
LC-MS/MS	Liquid chromatography–tandem mass spectrometry
MCO	Medium cut-off
MCO-HD	Medium cut-off hemodialysis
MW	Molecular weight
OH	Overhydration
OL-HDF	Online hemodiafiltration
P	Serum phosphate
PBUTs	Protein-bound uremic toxins
PCS	p-cresyl sulfate
Qb	Blood flow rate
Qd	Dialysate flow rate
RM ANOVA	Repeated measures analysis of variance
SCFA	Short-chain fatty acid
SPSS	Statistical Package for the Social Sciences
SV	Substitution volume
TMAO	Trimethylamine-N-oxide
